# Applying Blockchain Technology to Address the Crisis of Trust During the COVID-19 Pandemic

**DOI:** 10.2196/20477

**Published:** 2020-09-22

**Authors:** Anjum Khurshid

**Affiliations:** 1 The University of Texas at Austin Austin, TX United States

**Keywords:** blockchain, privacy, trust, contact tracing, COVID-19, coronavirus

## Abstract

**Background:**

The widespread death and disruption caused by the COVID-19 pandemic has revealed deficiencies of existing institutions regarding the protection of human health and well-being. Both a lack of accurate and timely data and pervasive misinformation are causing increasing harm and growing tension between data privacy and public health concerns.

**Objective:**

This aim of this paper is to describe how blockchain, with its distributed trust networks and cryptography-based security, can provide solutions to data-related trust problems.

**Methods:**

Blockchain is being applied in innovative ways that are relevant to the current COVID-19 crisis. We describe examples of the challenges faced by existing technologies to track medical supplies and infected patients and how blockchain technology applications may help in these situations.

**Results:**

This exploration of existing and potential applications of blockchain technology for medical care shows how the distributed governance structure and privacy-preserving features of blockchain can be used to create “trustless” systems that can help resolve the tension between maintaining privacy and addressing public health needs in the fight against COVID-19.

**Conclusions:**

Blockchain relies on a distributed, robust, secure, privacy-preserving, and immutable record framework that can positively transform the nature of trust, value sharing, and transactions. A nationally coordinated effort to explore blockchain to address the deficiencies of existing systems and a partnership of academia, researchers, business, and industry are suggested to expedite the adoption of blockchain in health care.

## Introduction

In many ways, it is hard for modern people living in First World countries to conceive of a pandemic sweeping around the world and killing millions of people, and it is even harder to believe that something as common as influenza could cause such widespread illness and death.

**Charles River Editors, *The 1918 Spanish Flu Pandemic: The History and Legacy of the World’s Deadliest Influenza Outbreak***

### The COVID-19 Pandemic in the United States and Worldwide

By the end of July 2020, COVID-19 had infected approximately 19 million people worldwide and had caused over 700,000 deaths. The United States is the richest country in the world, with a health expenditure of US $3.5 trillion per year; it has reported the highest number of people infected with COVID-19 (approximately 5 million) as well as the highest number of deaths (>150,000) [1]. Through a lack of reliable data, inability of health care and public health systems to perform active surveillance, inadequate management of needed medical equipment, conflicting information from multiple sources, and limited technology for engagement with patients, the COVID-19 pandemic has clearly demonstrated the failure of existing institutions to protect human health and to avoid widespread suffering.

### COVID-19 Is a Crisis of Trust

In the absence of reliable data and accurate information, the suffering due to the COVID-19 crisis has been exacerbated by misinformation, which ranges from warnings of imminent doom to conspiracy theories. The COVID-19 crisis is an information crisis [2]. This crisis has been rightly described as an “infodemic,” a term coined in 2002 by Eysenbach [3]. However, the importance of this term has been manifested in this crisis more than ever before by the enormous influence of social media and its role as a source of information for the public about the pandemic [4]. Several months into the greatest public health disaster in a century, with all our technology and information networks, there is still much confusion about the actual prevalence of COVID-19, number of deaths, expected treatments, and best strategies to control the pandemic globally [5,6]. Due to this failure to provide timely, accurate, and reliable information about the infection, the pandemic has worsened the crisis of trust in government institutions and public health agencies [7].

Interestingly, a similar failure of governmental response led to the 2008 financial crisis and precipitated the low trust in government and centralized institutions that has since persisted. Banks, governments, and financial institutions failed the public and left many people exposed and dejected during the financial crisis [8]. This motivated Satoshi Nakamoto, the pseudonym of an unidentified person or group, to write a paper that proposed a new system of establishing trust in financial markets without intermediaries such as governments or banks. This system relied on a distributed ledger technology of peer-to-peer networks and was called blockchain [9]. In 2009, Bitcoin was launched as a decentralized cryptocurrency that bypassed intermediaries such as banks, governments, and large financial institutions and allowed people to transact directly in a secure trust framework [10]. The COVID-19 pandemic promises to inflict even greater hurt and misery to the public than the 2008 financial crisis because it is not only an economic disaster but also a health calamity that has already caused the loss of precious lives worldwide. The role of intermediary organizations has also been unsatisfactory in this situation [11].

### Issues of Trust With Existing Institutions

The proponents of the decentralized and distributed system of blockchain technology point to the disadvantages of centralized institutions because these “intermediaries of trust” are slow to respond to changes in the environment, add cost and time to transactions, and adversely affect productivity [12]. Centralized institutions also store data centrally and not only restrict access to these data, thus preventing coordination and efficient sharing of information, but also represent a single point of failure for privacy and security of the information [13]. In 2017, the personal data of more than 143 million customers were exposed through a single breach [14]. According to one source, in the last 10 years, more than 1.4 billion records have been exposed due to government database breaches worldwide [15].

Intermediary institutions are supposed to provide much-needed, trustworthy, and reliable services to society [16]; however, the COVID-19 crisis has exposed the limitations of these institutions with regard to health care [17]. In this time of crisis, both public and private institutions as well as traditional information systems have mostly failed to solve problems related to routine health care delivery [18], including availability of timely data for projections of case numbers [19], identification of high-risk populations [20], tracing contacts of persons with COVID-19 [21], and supply of personal protective equipment (PPE) or inventories of lifesaving drugs [22]. In fact, it has been argued that the number of deaths due to COVID-19 could have been reduced with better access to reliable data [23].

### Blockchain and the “Trustless” System

Blockchain has been described as a foundational technology [24] that can dramatically change the paradigm in which social and economic transactions take place. A review of the key characteristics that form the fundamental aspects of blockchain technology may help demonstrate why this technology can be invaluable in addressing some of the issues of mistrust described above. Blockchain technology is based on a “trustless” system, where transactions can be performed among people who do not have any prior relationship yet who can validate the objectivity and principles of the medium in which the transactions occur. This enables transparency of contracts, immutability of data, and accountability of transactions among strangers [25]. Public blockchain networks allow individuals to share their information in complete privacy while maintaining full control of that information. They can also maintain an audit record of each transaction, make it readily available when needed, validate information sources to avoid misinformation, allow tracking of assets as part of the architecture of the network, and provide global connectedness without barriers to flow of information [26,27]. The rules of consensus and validation are transparent, mathematically proven, unbiased, distributed, and objective in nature; these characteristics cannot be ascribed to government or to most key institutions that are handling private information related to the pandemic [28]. The growth of the cryptocurrency market provides proof that the trustless system is a workable solution at a global stage; the COVID-19 pandemic has highlighted the necessity for such a solution [29].

## Blockchain in the COVID-19 Pandemic

As a foundational technology that promises to provide new solutions to old problems, blockchain technology is increasingly being applied in innovative ways that are relevant to the challenges created by the COVID-19 pandemic. The failure of existing systems to provide reliable and effective solutions to problems created by this global crisis has highlighted the potential of blockchain applications even more greatly [30]. The crisis has created a unique opportunity to test and develop blockchain-based solutions. It is difficult for health care organizations to implement blockchain technology and adopt its more open, transparent, patient-focused, and robust systems of transaction and information management without more evidence of its effectiveness; however, it is worth testing this technology to develop systems with levels of robustness that current information systems have not been able to achieve. Fortunately, there are already some use cases of blockchain technology that may significantly contribute more effectively to the fight against the COVID-19 pandemic and infodemic in the short run and build capacity to respond to similar health emergencies in the future. We discuss two key examples that relate to medical care directly and where blockchain technology is currently being applied but should be adopted even more widely if proven effective.

### Supply Chains and Blockchain

During the COVID-19 crisis, major supply chain failures have been observed not only for household items such as toilet paper and hand-washing soaps [31] but, more importantly, for PPE and lifesaving ventilators in hospitals and clinics [32]. Blockchain technology provides immutable and distributed ledgers with auditable records, which are ideal for tracking each asset in a supply chain because every actor in the supply chain shares the same information [33]. It is therefore easy to calculate the inventory and the exact stage where assets are in the chain; instant reconciliation can then be achieved without any additional audit or negotiation among the various suppliers and end users. A joint Walmart-IBM project demonstrated how tracking sources of contamination in green vegetables, a task that previously took months, could be achieved within seconds using blockchain [34,35]. Some of the lessons learned from that system are now being applied at the US Food and Drug Agency for counterfeit pharmaceuticals [36]. IBM also designed Rapid Supplier Connect to help with medical supply chains during the COVID-19 pandemic and offered it to health systems and government agencies to help find vendors for medical supplies and PPE [37].

**Figure 1:**
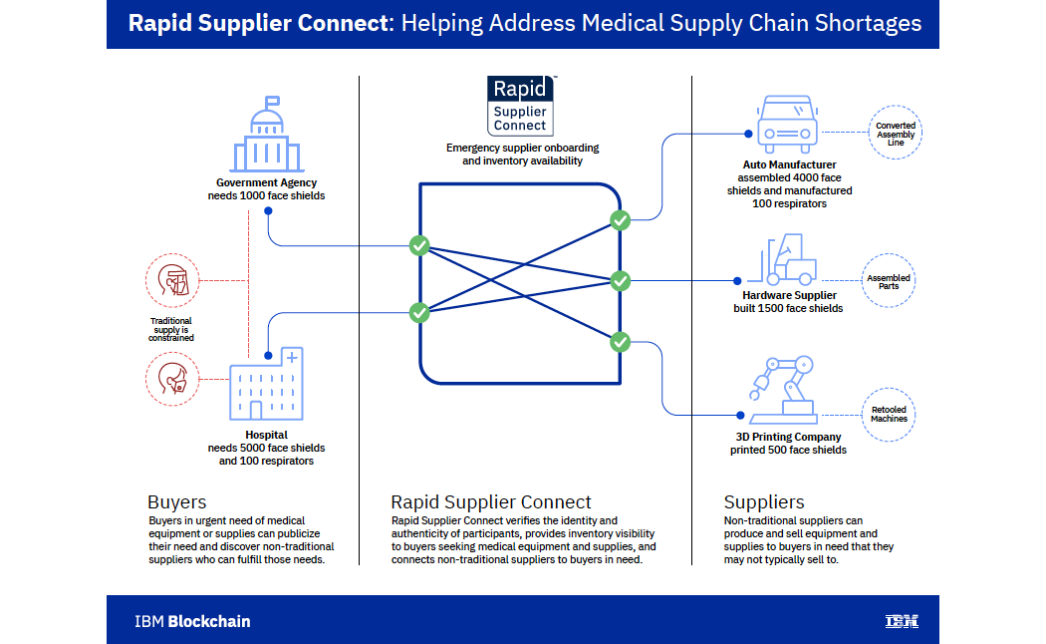
Schematic of IBM Rapid Supplier Connect [44].

Even during this global crisis, there have been reports of counterfeit medications and poor-quality equipment being sent to organizations and people who are in desperate need of these items [38]. This has led to trust issues in the supply chain [39]. In fact, a report by the Organisation for Economic Co-operation and Development cautioned about increased global trade in fake pharmaceuticals during the COVID-19 pandemic [40]. Blockchain not only provides an efficient way to manage the supply chain but also provides a means to distinguish quality products from counterfeit ones [41]. This is particularly true when items must be moved across international borders, where the levels of information about sources of production and the rules under which the quality checks occur vary greatly. Validation of the quality through peer-to-peer networks such as blockchain can improve trust and decrease unnecessary litigation and disputes [42,43]. An example of such a system is IBM’s Trust Your Supplier solution, in which blockchain enables trusted sources of supplier information and digital identity management to reduce the risk of counterfeiting while facilitating onboarding of suppliers and communications between buyers and sellers or suppliers and distributors [44]. As shown in Figure 1, Trust Your Supplier is a permissioned network that limits access to the information on the blockchain and allows for transparency among nodes on the supply chain. Cryptographic security ensures confidentiality of data on the chain, and the immutability of records guarantees that no one party can make changes unilaterally without a consensus.

**Figure 2:**
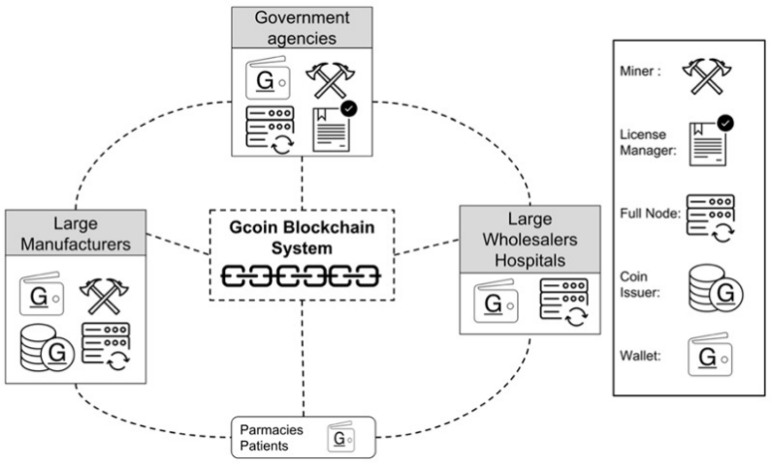
Schematic of the Gcoin blockchain system [45].

Another example of the use of blockchain to address the issue of counterfeit drugs is Gcoin (Figure 2) [45].

By maintaining the suppliers and buyers on a shared ledger (ie, blockchain) and by recording all transactions on the chain as immutable records, it becomes much easier to quickly see the origin of the products and also to search for the total supplies of an item without creating a centralized database. Centralized databases are not only difficult to keep up-to-date during crises but may also present easy targets for hacks and breaches. The trustless system of blockchain can significantly help with reducing supply chain failures, particularly as the pandemic enters the stage where vaccines and lifesaving drugs will need to move across international borders to save lives.

### Contact Tracing and Blockchain

For a highly infectious disease such as COVID-19, the ability to promptly trace individuals who have been exposed to an infected person is a beneficial public health strategy that can limit the continuing spread of the infection. As the number of COVID-19 cases continues to rise and surges are occurring worldwide, there is increased realization that social distancing and lockdown measures cannot be indefinitely extended. In many cases, compliance with lockdowns has been difficult to enforce, and coercion and significant resource allocation are needed to enforce it [46]. Scenes of police, army, and other government agencies roaming the streets to enforce closure of businesses and lockdowns are ubiquitous on the internet, with many examples of use of violence and threats against citizens [47]. With no other remedy to control the spread of the infection, widespread social distancing and lockdowns are blunt policy levers that are being used by most governments. However, the devastating consequences of this policy on economic activity, social interactions, mental health, and health-seeking behaviors are already visible, leading to the realization that this is not a sustainable strategy. Managing infections at an individual level and taking precautions in close circles that are at risk of infection will be needed to allow some level of normalcy to return. Contact tracing is an important tool in this regard [48].

Many states in the United States and countries worldwide have quickly developed and adopted contact tracing applications since the beginning of the pandemic, with mixed success [49]. As health care delivery systems were overwhelmed with testing and treatment needs, symptom-tracking apps were used to help individuals assess their risk of COVID-19 and their need for testing. Most of these apps require notifications or data sharing with public health or health care organizations to coordinate testing and treatment. With limited supplies of tests, only people with symptoms are asked to be tested. If an individual tests positive for COVID-19, the next steps are tedious public health investigations to identify who else is at risk of being infected by the individual. Traditionally, public health agencies have used contact tracers, who provide this information to potential contacts by telephone or mail and ask them to be tested. This approach is successful when the numbers are not of the magnitude seen in the COVID-19 pandemic. Also, these strategies were developed in the pre–mobile phone era. Due to the relative ease of mobile app development and ubiquitous access to mobile phones, contact tracing apps for COVID-19 are an obvious public health solution.

Countries such as Norway, South Korea, China, Singapore, Germany, and Qatar have developed, encouraged, or enforced the use of contact tracing apps as a public health strategy. Different technologies have been used to provide contact tracing; these technologies use various features built into mobile phone platforms, such as GPS, Bluetooth, Wi-Fi, and quick response (QR) codes. Very quickly, however, individuals and advocacy groups raised concerns regarding data privacy, confidentiality, and security [50]. Simultaneously, numerous reports of hacks, bugs, and misuse of data started to emerge. Some of these apps have been banned or discontinued. This lack of trust is not only reserved for governments but is also the reason that the Google-Apple contact tracing features have been regarded with concern in England and other countries [51]. However, the need for contact tracing and controlling infection by identifying people at risk has not decreased. This is a situation where the trustless system of blockchain technology can provide potential answers on how to balance public health needs with privacy concerns [52].

Blockchain enables information to be collected from individuals without identifying them by using a system of public and private keys [53]. For example, the BeepTrace system uses blockchain to provide encrypted and anonymized personal identification while allowing regulators and health care providers to contact people at risk of infection due to contact with an infected person. The system uses two chains and a public key generated by the government or a public entity to generate location data but also generates a diagnostician key to verify test results. The infected person gives consent to the diagnosing entity, which participates in the blockchain to verify results; however, the government cannot identify the individual. Notifications can be sent to the individual using a separate chain [54].

Previously, the same privacy and data sharing scheme was also proposed in other blockchain-based applications [25]. The key is that through anonymizing and cryptography, a blockchain-based contact tracing app ensures individual privacy while allowing public health departments to contact people who may have been exposed to SARS-CoV-2, the virus that causes COVID-19, through an infected person. These features of security, privacy, trust, transparency, and efficiency are built into the architecture of blockchain and have been difficult to replicate or develop reliably in other applications.

Countries such as Taiwan and South Korea have shown that a robust system of contact tracing can control the spread of infection while allowing normal life to continue for healthy people who are at low risk for infection [55]. However, concerns about privacy and security may limit the implementation of such strategies in different parts of the world, particularly in the United States, which has the highest numbers of cases and deaths [56]. Blockchain technologies that enable individuals to share their personal information in a secure manner with public health agencies without revealing their identity or contributing that information to a centralized government or corporate database may help identify people who come into contact with a patient who has tested positive for COVID-19. This can be achieved through public health agencies or through peer-to-peer notifications, where only the positive status can be shared without sharing other medical or personal data [57]. The capability to track individuals who are positive for COVID-19 and to check their seropositive status for infection may be used as a key tool to enable more responsible reopening of the economy without causing a surge in cases. As we develop vaccinations or develop herd immunity for the infection, blockchain technology may also be used to issue health certifications that can be verified easily by employers and public health agencies to validate the status of an individual [58].

Blockchain technology may be applied in many other aspects related to the long-term fight against the COVID-19 pandemic, such as approval of insurance status within seconds rather than the multiple contacts needed today to verify insurance [59], patient identification at the point of care that does not require filling out multiple forms and carrying documents to appointments with physicians [60], or conducting research without increasing the risk to privacy of individuals [61]. Artificial intelligence [62], the Internet of Things [63], and 3D printing [64] using immutable and verified instructions through blockchain are other technologies that may be greatly helpful in fighting pandemics such as COVID-19 in the future.

## Future Considerations

The devastation and suffering caused by the COVID-19 crisis should trigger a resolve to build better systems of data, trust, and transactions to track, respond, and control such pandemics in the future. While blockchain technology holds great promise, and solutions to systemic failures of our current health care, public health, and policy institutions are already being developed, the widespread adoption of this technology requires planning and execution. A national policy agenda to immediately consider how blockchain applications may help enable safe and effective responses to the COVID-19 pandemic will help expedite the acceptance, adoption, and implementation of this technology to improve our flawed systems of health care data and health-related transactions.

Major blockchain and software companies are already in the process of creating a decentralized governance system to create international standards, such as World Wide Web Consortium (W3C) and internet protocols. Hyperledger, Ethereum, BankChain, and R3 are all examples of such consortium-building efforts [65]. Consensus on protocols and rules of business among competitors and collaborators leads to more effective, egalitarian, and implementable rules, which have helped improve the interoperability and scalability of wireless and internet technologies. If governments and large private corporations actively participate and encourage this collaborative global governance rather than considering it a threat to their own hegemonic authority, health systems worldwide will be much better prepared for future health crises such as the COVID-19 pandemic.

Finally, research and development is needed to build and test robust use cases for blockchain applications. University and research institutions should partner with industry and business. Such collaborations are rare and must be established widely to expedite the adoption of blockchain technologies in health. Development and funding of blockchain implementation laboratories in universities and medical schools will help promote such industry-academia partnerships and provide stronger and more reliable evidence to evaluate the impact of blockchain technology in health care. Both health care and blockchain technology require interdisciplinary teams to work together to solve problems. Blockchain laboratories in academic medical centers and public universities can provide platforms for cooperation and creative problem-solving that will help in the fight against pandemics such as COVID-19.

## Conclusion

Blockchain technology relies on a distributed, robust, secure, privacy-preserving, and immutable record-keeping framework that can positively transform the nature of trust, value sharing, and transactions. The COVID-19 crisis has highlighted the failure of current systems of trust and data sharing. While this pandemic presents a clear and serious danger to our way of life, it also provides unique opportunities to apply and test new technologies that may help transform our capabilities to fight this pandemic and, in the process, establish a more efficient, democratic, and secure system to respond to future pandemics.

## Abbreviations

PPE: personal protective equipment
QR: quick response
W3C: World Wide Web Consortium
